# Physician Attitudes on the Use of Smartwatch Cardiovascular Data in Patient Care

**DOI:** 10.1016/j.jacadv.2025.101824

**Published:** 2025-05-30

**Authors:** Sajan Shroff, Emily Draper, Michael Massoomi, Phuong Huynh, David E. Winchester

**Affiliations:** a College of Medicine, University of Florida, Gainesville, Florida, USA; b Division of Cardiovascular Medicine, Department of Medicine, University of Florida, Gainesville, Florida, USA; c Office of Student Affairs, College of Medicine, University of Florida, Gainesville, Florida, USA

**Keywords:** ambulatory cardiovascular data, Apple watch, digital health, smartwatch, technology

**What is the clinical question being addressed?**

What are the physician-perceived barriers to integrating smartwatch cardiovascular data into patient care?

**What is the main finding?**

The primary barriers identified are time constraints, limited reimbursement, and likely misplaced concern regarding the accuracy of single-lead ECG.

Smartwatches are increasingly valuable tools for physicians to monitor patient cardiovascular health, generating large amounts of biometric data. As clinicians encounter more smartwatch collected data, many have developed opinions on its benefits and limitations in patient care. Current research focuses on the accuracy of detecting arrhythmias like atrial fibrillation,^1^ and using heart rate (HR) data to assess stress.^2^ While one study examined physician responses to hypothetical Apple watch-detected atrial fibrillation alerts,^3^ few have explored overall physician attitudes toward smartwatch data. We sought to assess physician perspectives on the current uses of smartwatch data in patient care and whether these opinions varied across demographics.

## Methods

We conducted an email-based survey of physicians in family medicine, internal medicine, and cardiology at the University of Florida. The survey evaluated physician attitudes on the following using a five-point Likert scale: the accuracy of smartwatch data (step count, HR, electrocardiogram [ECG]), comfortability counseling patients based on smartwatch data, and current shortcomings. Respondents were asked to rate statements from 1 to 5 where: 1 = “strongly disagree,” 2 = “somewhat disagree,” 3 = “neither agree nor disagree,” 4 = “somewhat agree,” and 5 = “strongly agree.” There were additional sections for demographics and an open response question at the end of the survey asking for any opinions not addressed.

The survey format was validated using a pilot study of 15 physicians, 10% of our target population, which yielded a Cronbach’s alpha of 0.782 (0.668, 0.896), providing validity to our survey. We estimated sample size by the number of clinical faculty and residents within the aforementioned departments at University of Florida. The survey was then sent via listserv to residents, fellows, and attending physicians.

Descriptive statistics and analysis of variance (ANOVA) were done in IBM SPSS Statistics (Version 29.0.2.0) using a *P* value of 0.003 to correct for false positives using the Bonferroni correction. During ANOVA testing, demographic groups were adjusted to improve validity of analysis.

## Results

We distributed our survey to 256 physicians, which yielded 79 completed responses, a 30.8% response rate. Our survey respondents were 57% (45/79) male (45/79) and 45% (36/79) of them were of ages 30 to 39 years. Sixty-five percent (51/79) of respondents were at the resident level, and 71% (56/79) of respondents practiced internal medicine and related subspecialties (noncardiology) as compared to 9% (7/79) practicing family medicine and 20% (16/79) cardiology. In terms of practice setting, 33% (26/79) of respondents practiced primarily outpatient medicine, 24% (19/79) did even amounts of inpatient and outpatient, and 43% (34/79) practiced mainly inpatient. Survey statement response results are summarized in Table 1.

**Table 1:** Summary of Statements With Mean Likert Scale Score

Statement	Median	IQR
I believe checking smartwatch data is valuable to patient care.	4.00	0.75
I trust the accuracy of my patient’s step count provided by their smartwatch device.	4.00	0.00
I trust the accuracy of my patient’s HR data provided by their smartwatch device.	4.00	0.75
I can understand and interpret smartwatch step counts and how it correlates to my patient's overall health and prognosis.	4.00	1.00
I can understand and interpret smartwatch heart rate data (resting HR, exercising HR, HR recovery, etc) and how it correlates to my patient's health.	4.00	1.00
I feel comfortable clinically interpreting step count data to make recommendations to improve my patients' health and prognosis.	4.00	2.00
I feel limited by time during patient visits to examine smartwatch collected health data.	4.00	2.00
I can understand and interpret smartwatch ECG data and how it correlates to my patient's health.	4.00	2.50
I feel comfortable in my ability to locate health data on a patient’s smartwatch or phone.	4.00	3.00
I feel comfortable asking for and using my patient’s phone and smartwatch to extract data.	3.00	2.00
I feel comfortable giving my patients recommendations and ordering additional tests/follow-up when needed based on smartwatch HR data.	3.00	2.00
I feel comfortable giving my patients recommendations and ordering additional tests/follow-up when needed based on smartwatch ECG data.	3.00	2.00
I trust the accuracy of my patient’s ECG data provided by their smartwatch device.	3.00	2.00
I feel comfortable with charting/logging smartwatch data into my electronic medical record.	2.00	2.00
I feel comfortable applying appropriate billing codes to compensate for time spent evaluating smartwatch data.	1.00	1.00

ECG = electrocardiogram; HR = heart rate.

Overall, respondents agree that checking smartwatch data is valuable to patient care (median = 4). They also trust the accuracy of wearable HR (median = 4) and step count data (median = 4) more than ECG data (median = 3). Respondents feel uncomfortable applying billing codes to compensate for their time spent reviewing data (median = 1) and are unsure about how to log data into electronic medical record (EMR, median = 2).

ANOVA testing was performed comparing respondent age (<29, 30-39, 40+), gender, (male, female, unspecified), training level (attending, trainee), and practice setting (mainly inpatient, mainly outpatient, mixed). There were no statistically significant differences in responses between each of these groups for each of the statements in Table 1.

## Discussion

Through our survey, we characterized current physician perceptions on wearable cardiovascular data. Overall, physicians agree that using these data is valuable in clinical care; however, there is less confidence in the accuracy of single-lead ECG compared to other metrics. Major barriers cited include the lack of billing codes and limited time during clinical encounters.

Identifying outlier perceptions through this survey can help reveal barriers to integrating wearable data into clinical practice. It is notable that the lowest scoring question was regarding billing and compensation with physicians simultaneously also agreeing that they feel limited by time during clinical visits. Given the growing demand for care and limited clinician time, it is unsurprising that clinicians cite appropriate compensation for time as a main area of concern. Creating new billing codes for wearable interrogation and increasing the available time to provide individualized care could result in more use of smartwatch data.

Another question that scored low was regarding clinician comfort in asking for a patient’s phone to view data, suggesting concern over privacy. Keeping the phone on the table so patients can watch along can help maintain transparency in the data review process. Also scoring low was comfort in charting smartwatch data into the EMR. Improvement in integration of data systems directly into existing EMRs in the future may help reduce the burden of documentation while also reducing the perceived “invasiveness” of using patient devices.

Step count emerged as the most trusted metric, with the highest median score and no variability. However, wider IQRs for understanding its health relevance and making recommendations based on it highlight a knowledge gap potentially limiting its clinical utility. Another notable finding is that respondents reported greater trust in HR data over ECG. While this study does not evaluate the accuracy of these metrics, existing research supports the accuracy of single-lead ECG, despite limitations compared to a traditional 12-lead ECG.^4^ In contrast, HR data can have a wider margin of error—likely around 10% to 20% at higher HRs.^5^ These responses suggest that clinicians may benefit from education regarding the accuracy and limitations of smartwatch data metrics.

Survey results were limited by sample size and should be repeated with a larger population to increase study power. Moreover, a large percentage of our respondents were trainees, biasing our results away from physicians with extensive clinical practice in the outpatient setting. Future studies may focus on analyzing qualitative open-ended responses from these physicians with outpatient practices.

## Conclusions

This survey demonstrated that while physicians believe that smartwatch data are useful to clinic practice, barriers remain. The primary barriers identified by this survey regarding smartwatch use in clinical care are time constraints, limited reimbursement, and likely misplaced concern regarding the accuracy of single-lead ECG. The creation of new billing codes for “wearable health device interrogation” could help lead to increased use of smartwatch data. The ultimate clinical impact of using such data warrants independent study.

## Funding support and author disclosures

The authors have reported that they have no relationships relevant to the contents of this paper to disclose.
